# Supplementary material for: Pathogenic and Prognostic Roles of Paraneoplastic Leukocytosis in Cervical Cancer: Can Genomic-Based Targeted Therapies Have a Role? A Literature Review and an Emblematic Case Report
Source: Diagnostics (Basel). 2022 Aug 7;12(8):1910. doi: 10.3390/diagnostics12081910 (PMC9406983; doi:10.3390/diagnostics12081910)
Supplement: Supplementary file 1 [file diagnostics-12-01910-s001.zip › diagnostics-1788850-supplementary.pdf]

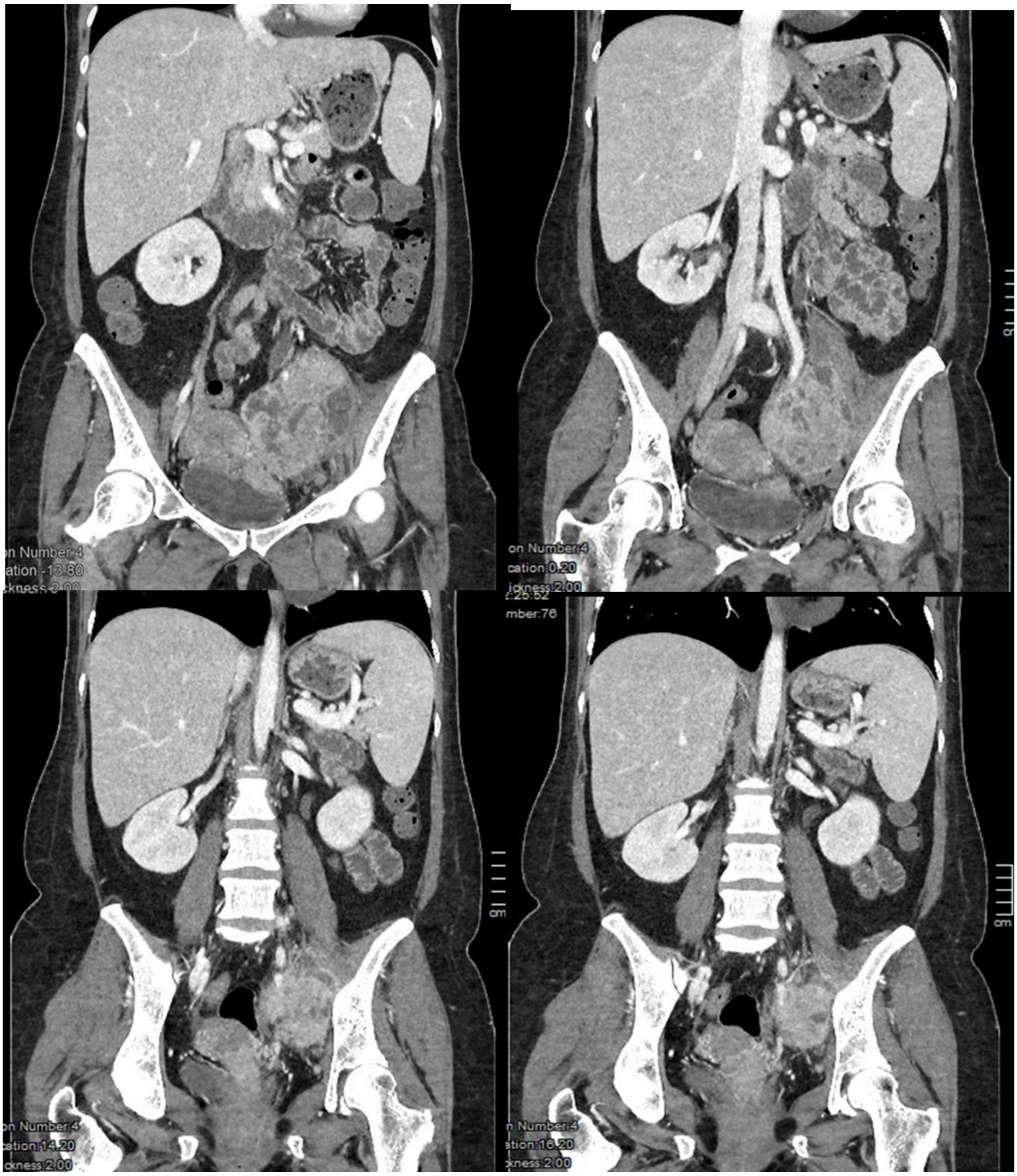

Supplementary Figure S1. CT assessment before cytoreductive/palliative surgery. CT coronal view showing the primitive neoplastic lesion infiltrating the bladder, necrotic colliquative adenopathies in the external and internal left iliac, and a voluminous secondary mass in the context of the left iliopsoas muscle with areas necrotic in the context (108×85×80 mm). A severe bilateral hydroureteronephrosis with the left ureter that appeared incorporated in the mass in its distal tract is observed

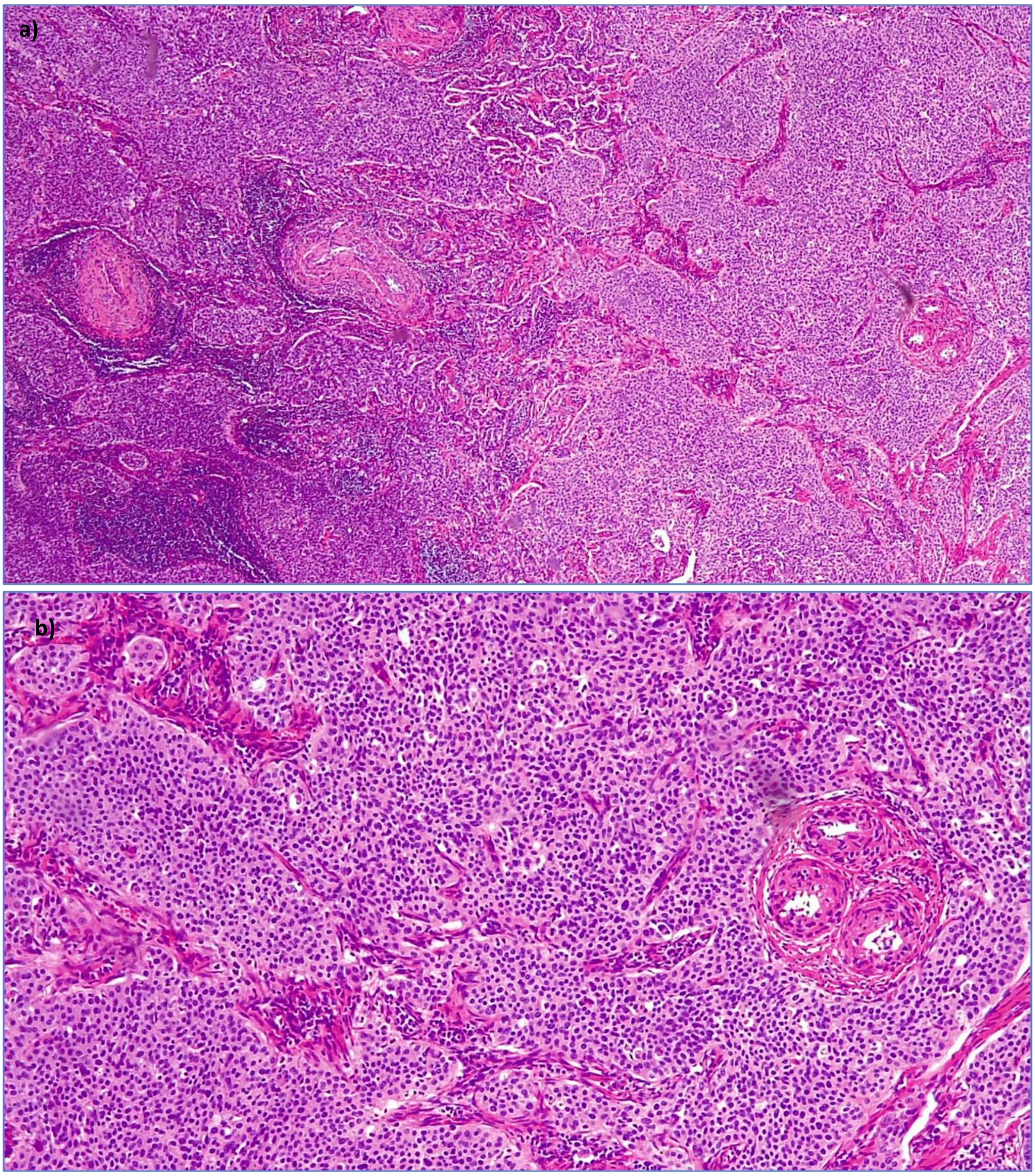

Supplementary Figure S2. Histological examination at hematoxylin eosin at 100X (a) and 200X (b) showing a squamous cell cervical carcinoma of the cervix, grading 2-3, with necrosis.

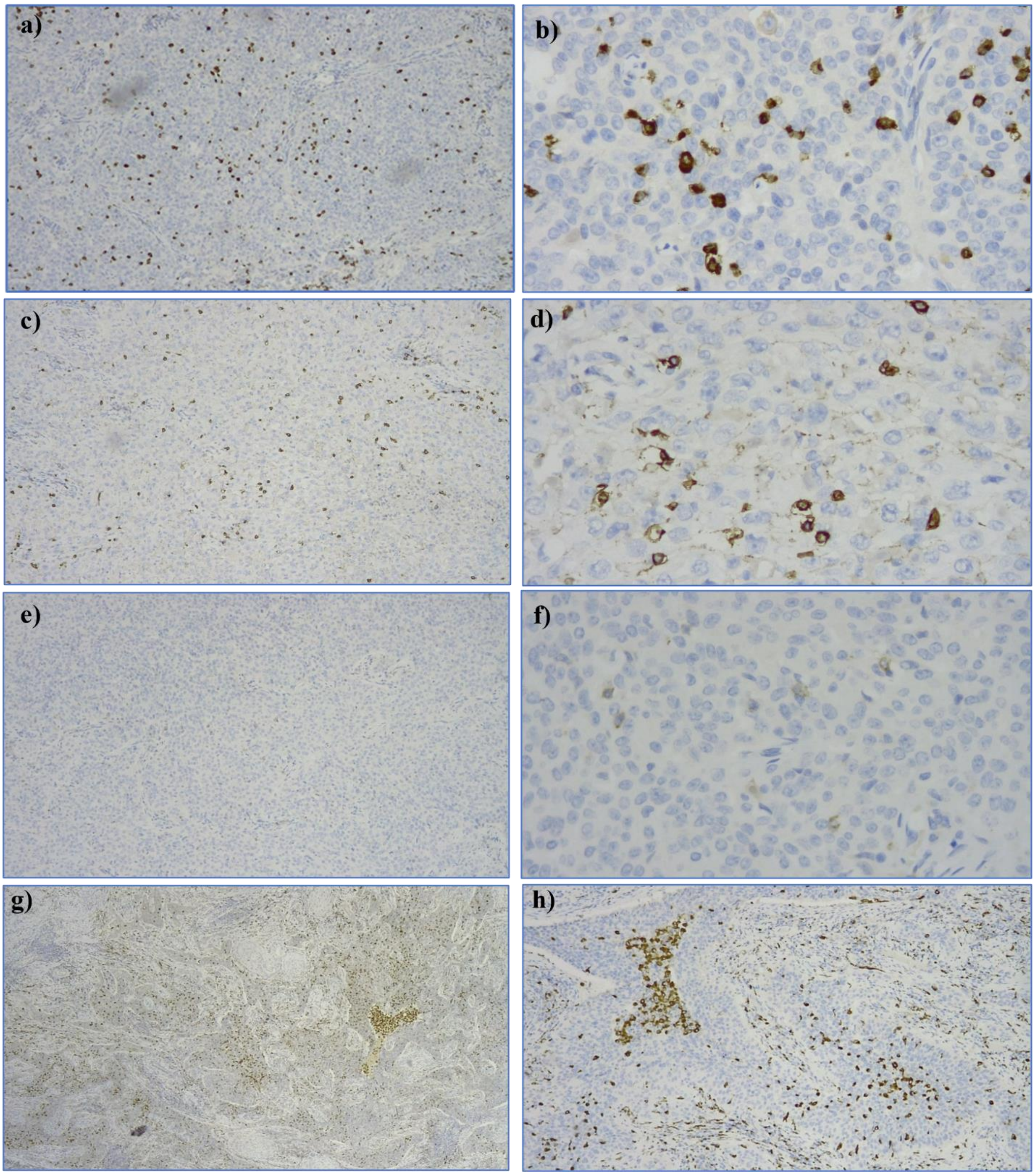

Supplementary Figure S3. Intratumoral lympho-monocytic and granulocytic infiltrate. The intratumoral lymphomonocyte and granulocytic infiltrate comprises: - frequent T lymphocytes CD8+ (a 200x; b 400 x), CD7+ (c 200 x; d 400 x) and PD1+ (e 200 x; f 400x); - numerous macrophages CD68+ (g 200 x; h 400 x); - rare neutrophils CD15, MPO, CD33 positive; -scattered eosinophils (associated to necrotic areas).

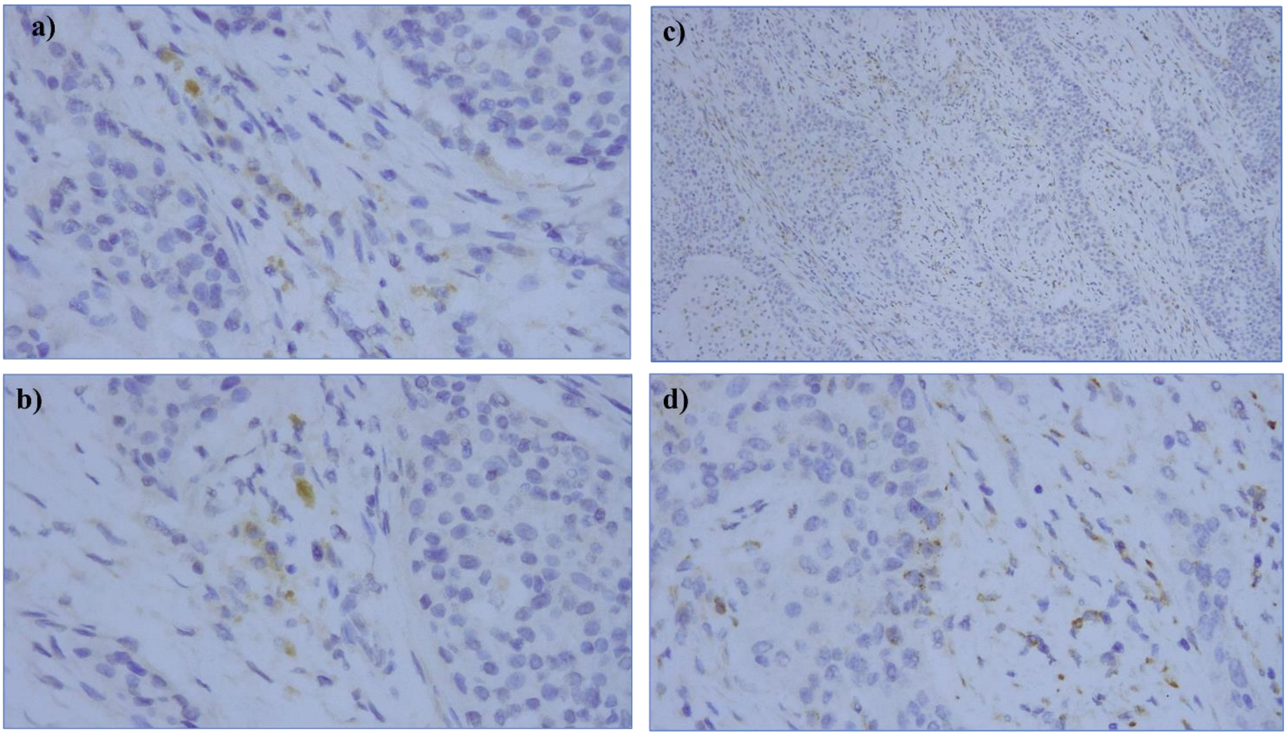

Supplementary Figure S4. HIC intratumoral and peritumoral staining of IL-6 and G-CSF. A-B) HIC (granular) cytoplasmatic positive staining of IL-6 in peritumoral macrophages (a) and cancer cells (b). 40x for both images. C-D) HIC intratumoral and peritumoral (granular) cytoplasmatic positive staining of G-CSF in cancer cells and macrophages at 20x (c) and 40 x (d) magnificence.
